# Multiphoton single-molecule localization by sequential excitation with light minima

**DOI:** 10.1038/s41377-022-00763-2

**Published:** 2022-03-25

**Authors:** Luciano A. Masullo, Fernando D. Stefani

**Affiliations:** 1 Centro de Investigaciones en Bionanociencias (CIBION), Consejo Nacional de Investigaciones Científicas y Técnicas (CONICET), Godoy Cruz 2390, C1425FQD Ciudad Autónoma de Buenos Aires, Buenos Aires, Argentina; 2 Departamento de Física, Facultad de Ciencias Exactas y Naturales, Universidad de Buenos Aires, Güiraldes 2620, C1428EHA Ciudad Autónoma de Buenos Aires, Buenos Aires, Argentina; 3 Present Address: Max Planck Institute of Biochemistry, Am Klopferspitz 18, 82152 Martinsried, Germany

**Keywords:** Super-resolution microscopy, Multiphoton microscopy

## Abstract

Using sequential excitation with a minimum of light to localize single fluorescent molecules represented a breakthrough because it delivers 1–2 nm precision with moderate photon counts, enabling tracking and super-resolution imaging with true molecular resolution. Expanding this concept to multi-photon regimes may be a useful complement to reach even higher localization precision and get deeper into biological specimens.

More than 30 years ago, confocal fluorescence microscopes were used for the first time to detect single molecules^1,2^. Since then, single-molecule detection and spectroscopy have evolved into a variety of analytical and microscopy approaches that go beyond ensemble averages.

The process of inferring the position of a single fluorescent emitter from the detected signal is called *localization*. One prominent application is super-resolution fluorescence imaging by single-molecule localization microscopy (SMLM)^3–7^. Most commonly, SMLM is performed in wide-field microscopes using uniform illumination. A multitude of blinking fluorescent molecules are imaged individually in a series of acquisitions with a photodetector array (e.g., an EM-CCD or CMOS camera), and their positions are obtained from a fit to the registered signals. The localization precision, and thus the resolution of these techniques is limited by the photostability of the fluorophores^7–9^. Typically, for organic fluorophores under biologically compatible conditions, this approach delivers a lateral localization precision in the range of 10–50 nm.

MINFLUX^10^ represented a breakthrough in single-molecule localization and super-resolution imaging because it provides a ~10-fold improvement compared to wide-field camera-based localization, reaching 1–2 nm precision with moderate photon counts, enabling imaging with true molecular resolution. MINFLUX remains the most photon-efficient single molecule localization method and has been demonstrated in model systems (DNA-origami structures) and biological cells^10^, it was extended to three dimensions^11^, and combined with fluorescence lifetime measurements^12^. In a recent work in eLight^13^, Kun Zhao et al. have made the first step in extending MINFLUX to the multi-photon regime. Through semi-analytical calculations and simulations, they compute the Cramér–Rao bound of the localization error of two-photon MINFLUX (2p-MINFLUX) for different experimental parameters and study the details, advantages, and disadvantages of such an implementation in comparison to the one-photon counterpart (1p-MINFLUX).

Intuitively, there are at least three ways of grasping MINFLUX. It may be regarded as a kind of triangulation to determine the emitter position. From this point of view, the need for a fourth exposure in the center of the pattern is not intuitive but can be understood when looking at the behavior of the position estimator. Alternatively, it could be thought of as a combination of single-molecule localization with structured illumination microscopy (SIM) in the special case where the prior knowledge of having only one molecule in the emitting state is used to estimate the molecular position. Finally, it could be understood as a comparison between two “images” of the excitation field: (i) a very detailed image obtained in reference measurements and (ii) a sparse image obtained using the target single emitter as a probe over a smaller number of positions. The position of the molecule is inferred as the parameter that best matches the relative intensity of the single-molecule image in comparison to the reference image.

The findings by Kun Zhao et al. can be framed and understood intuitively from any of these points of view. MINFLUX benefits from the 2-photon excitation non-linearity just like confocal and other super-resolution microscopy methods do^14,15^: the quadratic dependence of the emission on the excitation intensity enhances the information content of the excitation scheme. Practically, it should also contribute to decreasing the background generated by autofluorescence and hence improve the signal-to-background ratio (SBR) of the measurement.

MINFLUX can also be thought of as part of a family of single-molecule localization methods that infer the molecular position from the signal registered upon excitation with a sequence of spatially shifted patterns of light. This type of method can be interpreted in a common framework^16^ and termed single-molecule localization by sequential structured illumination (SML-SSI). They were first developed for single-molecule tracking using Gaussian beams^17–22^, which were also applied with multi-photon excitation^23,24^. Conceptually, the breakthrough of the original MINFLUX work was two-fold. First, it demonstrated the advantage of exciting molecules with a minimum of light. Second, it combined the sequential structured illumination with single-molecule blinking to obtain super-resolved images. This had not been demonstrated by any of the previous SML-SSI methods, such as Orbital Tracking^17,25^, which had been applied exclusively for single-molecule/single-particle tracking.

The common framework of SML-SSI^16^ can be easily extended to multi-photon regimes. In the most general case, a sequence of exposures to a position-dependent excitation intensity *I*(***r*** − ***r***_**i**_), the “excitation pattern”, is used to infer the position of the emitter ***r***_**E**_. For each *I*(***r*** **−** ***r***_**i**_), the emitter is exposed to a specific local excitation intensity *I*(***r***_**E**_ − ***r***_**i**_) and emits fluorescence accordingly, which corresponds to an expected value of detected photon counts (*λ*_*i*_) during a given integration time. The measured fluorescence photon counts are denoted by *n*_*i*_, which are assumed to be Poisson distributed with average *λ*_*i*_. The position of the emitter is determined from the sequence of intensity measurements ***n*** = [*n*_1_, *n*_2_,…, *n*_*K*_], and considering the known *I*(***r*** − ***r***_**i**_). The relationship between *I*(***r***_**E**_ **−** ***r***_**i**_) and *λ*_*i*_ can be assumed to be linear but also of higher order due to the multiphotonic excitation, hence yielding, *λ*_*i*_ ∝ *I*^*c*^(***r*** **−** ***r***_**i**_). Where *c* is the order of the multiphotonic excitation process; e.g. *c* = 2 for two-photon, or *c* = 3 for three-photon excitation. Fractional exponents could also be used to represent more complex superlinear excitation schemes, such as when using photoswitchable fluorescent proteins^26,27^. Kun Zhao et al. study thoroughly the case *λ*_*i*_ ∝ *I*^2^(***r*** **−** ***r***_**i**_) for MINFLUX.

The potential advantages of using multi-photon excitation in SML-SSI are analogous to other microscopy methods. The out-of-focus background should be drastically decreased because of the reduced effective excitation volume. The possibility of using longer wavelengths provides a higher penetration depth which is particularly useful for biological tissues, and the superlinear dependence of the excitation intensity can be exploited to enhance resolution. Kun Zhao et al. explore in detail this latter aspect demonstrating a ~2-fold improvement in localization precision at the center of the excitation pattern. As in MINFLUX and every SML-SSI the localization precision increases following *σ* ∝ 1/√*N*, hence the benefit of 2p-MINFLUX can also be regarded as requiring 1/4 of detected photons from the one-photon counterpart.

It was recently shown that SML-SSI methods using minima of light share their photon efficiency, independently of the spatial arrangement of the excitation sequence^16^. A practical alternative to MINFLUX is to perform the localization using a raster scan of minima, in the so-called RASTMIN^16^ scheme, as it is done in any (linear or multi-photon) scanning microscope. Figure 1 shows example calculations of the Cramér–Rao Bound of the localization error (*σ*_CRB_) as a function of the emitter position for equivalent configurations of MINFLUX and RASTMIN under 1p-, 2p-, and 3p-photon excitation. In both cases, the superlinear excitation leads to higher localization precisions in the center of the excitation pattern. Remarkably, the RASTMIN scheme solves the issue pointed out by Kun Zhao et al. for 2p-MINFLUX that the precision becomes more heterogenous across the field-of-view defined by the excitation pattern. The calculations were performed using code that can be found at https://github.com/lumasullo/sml-ssi or https://github.com/stefani-lab/sml-ssi.

**Fig. 1:**
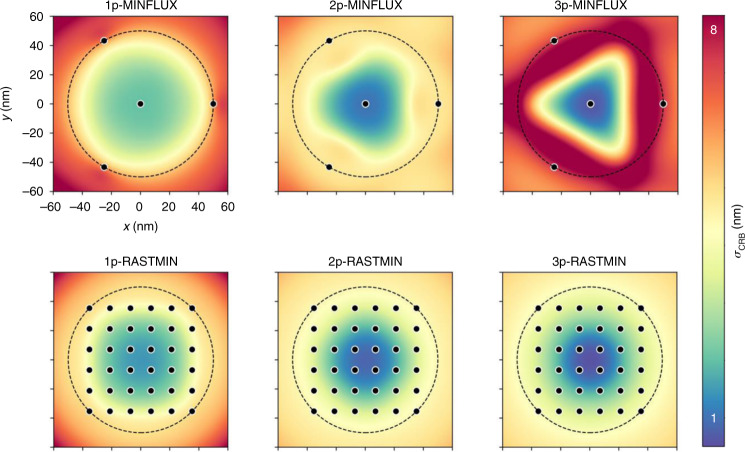
Precision maps (localization error lower bound *σ*_CRB_ as a function of the emitter position) for equivalent configurations of MINFLUX and RASTMIN, for 1-photon, 2-photon, and 3-photon excitation. Parameters: *L* = 100 nm, *N* = 500, SBR = 4, *λ* = 647, 800, and 1300 nm for 1-, 2- and 3-photon processes, respectively

SML-SSI approaches using minima of light enable single-molecule localization, tracking, and imaging of molecular dynamics with an unmatched spatiotemporal resolution, with impact in various fields, particularly biophysics and molecular nanophotonics. The two-photon MINFLUX studied theoretically by Kun Zhao et al. constitutes the first approach to extend such methods to the multi-photon regime, where advantages are shared with other multi-photon microscopy techniques, namely higher spatial resolution, reduced background, reduced photodamage, and enhanced penetration depth.

Finally, we make a few considerations for experimental implementations. First, it is remarkable that there are no serious challenges to implementing these methods in existing multi-photon microscopes, particularly in the form of RASTMIN. While it could be argued that a similar improvement in localization precision could be attained by reducing the field of view *L* in a 1-photon configuration, it should be noted that *L* cannot be reduced indefinitely in practice due to the drop in SBR. The super-linear excitation could provide an advantage in this respect. On the downside, the high light doses required for multiphoton excitation tend to lead to photobleaching, making single-molecule measurements difficult. Expanding the library of fluorophores with sufficient photostability and suitable blinking behavior under multiphoton excitation is an important experimental question. Alternatively, an effective non-linear excitation could be achieved through other photophysical processes. For example, photoswitching by *cis*/*trans* isomerization of fluorescent proteins results in a quadratic dependence with the illumination intensity^28,29^. Such schemes would have the advantage of using significantly lower light doses.
